# High Desalination Performance of Polyamide Composite Reverse Osmosis Membranes Based on Integrated Diamine Monomers

**DOI:** 10.3390/membranes16050163

**Published:** 2026-04-30

**Authors:** Caiyun Liu, Chen Chen, Wencai Zhang, Hongyang Ma, Shyam Venkateswaran, Benjamin S. Hsiao

**Affiliations:** 1 State Key Laboratory of Organic-Inorganic Composites, Beijing University of Chemical Technology, Beijing 100029, China; 2 Department of Chemistry, Stony Brook University, Stony Brook, NY 11794-3400, USAbenjamin.hsiao@stonybrook.edu (B.S.H.)

**Keywords:** integrated monomers, reverse osmosis membrane, interfacial polymerization, antifouling, desalination

## Abstract

Polyamide thin-film composite reverse osmosis membranes were fabricated through interfacial polymerization (IP), wherein trimesoyl chloride (TMC) and isomeric diamine monomers including *o*-phenylenediamine (OPD), *m*-phenylenediamine (MPD), *p*-phenylenediamine (PPD), and methyl-substituted monomers such as 2,3-diaminotoluene (MOPD), 2,4-diaminotoluene (MMPD), 2,5-diaminotoluene (MPPD), and 2,6-diaminotoluene (2,6-MMPD) were employed. The membranes with high permeation flux and rejection ratio were eventually applied in the desalination of brackish water. The regional effects of the amino and methyl substituent on the desalination performance of the RO membranes in terms of permeation flux and rejection ratio were investigated extensively. A molecular dynamics simulation based on the configuration of monomers was performed to theoretically explore the effects of amino and methyl groups of the monomer on the packing density of the aromatic molecular structure and, consequently, on the desalination performance of the corresponding RO membranes. The RO membranes with integrated monomers exhibited two times higher permeation flux than that of a pristine RO membrane while remaining the high rejection ratio. Moreover, a long-term desalination performance of the RO membrane was also demonstrated, where two times higher permeation flux than that of conventional and commercial RO membranes was achieved, while the rejection ratio was maintained at 97.6% which was comparable with that of the commercial RO membranes.

## 1. Introduction

With the world population booming and with rapid industrialization, the scarcity of freshwater supplies has become a global issue that needs to be addressed on a priority basis [1,2]. Against this background, brackish water desalination technology has become a crucial and efficient approach when it comes to addressing the freshwater supply–demand imbalance [3,4,5]. Thus, desalination of saline water with high efficiency could solve the problem of shortage of freshwater. Among different desalination approaches, including direct distillation [6], electrodialysis [7], membrane distillation [8,9], and forward osmosis [10,11], reverse osmosis (RO) membrane separation has been regarded as a more efficient, more energy-saving, and a significantly more cost-effective method for converting saline water to freshwater compared to other processes [12,13,14,15].

Typical reverse osmosis membranes represent thin-film composite (TFC) membranes that exhibit a three-layered structure: (1) a polyester nonwoven fabric for bulk mechanical strength (~120 µm), (2) a polymeric support layer (40–60 µm) synthesized via phase inversion, and (3) a thin polyamide selective layer (150–300 nm) [16,17,18,19]. In reverse osmosis membranes, the polyamide active layer plays a decisive role in the membrane’s permeability–selectivity trade-off, while the performance of the support layer also exerts a significant influence on water permeability—both layers are critical to the overall separation performance of the membrane [17,18,19,20,21,22,23]. Commonly used materials for the preparation of barrier layers on RO membranes include cellulosic derivatives [24,25,26], cross-linked polyamide [14], and other related polymers [27,28,29], inorganic materials [30], and other mixed matrix materials [31,32,33]. To date, polyamide TFC membranes are arguably the most promising materials for reverse osmosis (RO) desalination membranes owing to their high permeability and selectivity, excellent thermal and chemical stability, and a wide pH applicability range [25,34]. A polyamide reverse osmosis membrane is typically fabricated via the interfacial polymerization (IP) of two reactive monomers, trimesoyl chloride (TMC) and m-phenylenediamine (MPD), dissolved in mutually immiscible solvents, separately, on a porous ultrafiltration support, which results in a rapid formation of a highly cross-linked thin film with dense structures [35,36,37].

There has been considerable work focused on different monomers, interfacial polymerization parameters, and the permeance–selectivity trade-off. [38,39,40]. Current research topics are focusing on the relationship between the structure of the barrier layer and the filtration efficiency of a RO membrane through overcoming classic challenges, such as membrane fouling [41,42], chlorine tolerance [29,43], and low permeability and selectivity [44,45,46,47,48,49,50,51].

A variety of acyl chloride and isomeric diamine monomers were employed to build up a polyamide matrix, which has been used to serve as the barrier layer of RO membranes [52,53]. The structure of the polyamide barrier layer was adjusted by using different diacyl chloride and isomeric diamine monomers. As a result, the permeability and rejection ratios of the corresponding RO membranes, depending on the structure, have been explored. Moreover, special monomers, such as *m*-phenylenediamine-4-methyl (MMPD) and cyclohexane-1,3,5-tricarbonyl chloride (HTC), have been employed to fabricate RO membranes with improved chlorine tolerance due to the reduced probability of N-chlorination and Orton rearrangement [54]. Our recent work helped us discover the inherent mechanism regarding the correlation of polyamide structure and separation efficiency of the RO membrane [55]. We found that the packing approach of aromatic molecular motifs in the barrier layer directly affects the water permeability and salt rejection ratio. Aromatic molecular motifs in the polyamide barrier layer could pack together through parallel and perpendicular (T-shaped) approaches, depending on the fabrication process; this was observed due to the orientation structure of molecules in the polyamide matrix and was been evidenced by grazing-incidence wide-angle X-ray scattering (GIWAXS) measurements. The parallel packs of aromatic molecular motifs in the polyamide barrier layer create dense regions, which corresponds to the 3.5–4.0 Å “π-π spacing” of the molecular motif, while the perpendicular packs of the aromatic molecular motifs exhibit 5.0 Å packing spacing, and, therefore, lead to high water flux.

Encouraged by this finding, we chose isomeric diamine monomers, *o*-, *m*-, and *p*-phenylenediamine, as well as methyl-substituted *o*-, *m*-, and *p*-phenylenediamine and their integration, to explore the effects of the relative location of diamino and methyl groups on the permeation flux and rejection ratio of the RO membranes. The structural and morphological characteristics of the corresponding polyamide barrier layer were investigated through the SEM and AFM measurements. The integration of the isomeric diamine monomers offers higher permeation flux to the corresponding RO membranes when compared with that of the conventional RO membranes. The amino and methyl-substituted groups covalently linked to the benzene rings of the monomers, which directly changed the water permeability and rejection ratio of the RO membrane, as well as the antifouling properties of the conventional RO membrane. Moreover, the possibility of a practical desalination application was evaluated based on a long-term desalination performance.

## 2. Materials and Methods

### 2.1. Reagents

Trimesoyl chloride (TMC, >99%), six diamines, including *o*-phenylenediamine (OPD, AR, 98%), *m*-phenylenediamine (MPD, >99%), *p*-phenylenediamine (PPD, AR, 97%), 2,3-diaminotoluene (MOPD, 98%), 2,4-diaminotoluene (MMPD, 98%), and 2,6-diaminotoluene(2,6-MMPD), and bovine serum protein (BSA, molecular biology-grade) were purchased from Aladdin Reagent Company (Shanghai, China), while 2,5-diaminotoluene (MPPD) was extracted with dichloromethane (AR, Beijing Chemical Works, Beijing, China) from 2,5-diaminotoluene sulfate (Aladdin, 98%) neutralized by sodium hydroxide (AR, Beijing Chemical Works). All these amine monomers were purified through sublimation under reduced pressure before use. The support layer of RO membranes was a polysulfone-based ultrafiltration membrane (PS-35), which was purchased from Sepro America, LLC (Pittsburgh, PA, USA). Hexane (AR) was received from Beijing Chemical Works. Sodium chloride (AR, 99%) from Aladdin Reagent Company was used to prepare the feed aqueous solution with a concentration of 2000 ppm.

### 2.2. The Preparation of the RO Membrane

**Figure 1:**
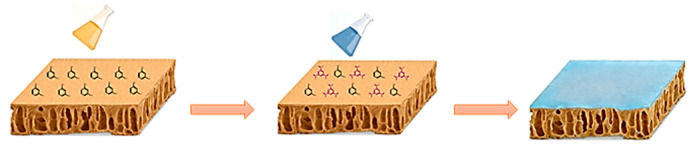
The scheme of the preparation of the RO membrane (Black represents aqueous-phase monomers, and purple represents organic-phase monomers).

The RO membranes were prepared through interfacial polymerization of TMC in hexane and MPD (or integrated diamine monomers) in water. Firstly, a PS-35 substrate membrane was taped onto a glass plate, followed by soaking the substrate with diamine aqueous solution (2.0 wt%) for 10 min. Then, the excess diamine solution was drained off from the surface of the PS-35 substrate. The organic TMC solution, prepared by dissolving TMC in hexane (0.1 wt%), was applied to the soaked substrate for 1 min to carry out interfacial polymerization, and an active skin layer over the surface of the PS-35 support was created, as shown in Figure 1. After removing the excess organic solution, the prepared membrane was heated at 80 °C for 10 min to allow hexane and water to evaporate, and then the membrane was stored in air overnight prior to use. The types and concentrations of monomers in the organic and aqueous phases are presented in Table 1.

**Table 1:** The reactions of various aqueous phase monomers with TMC.

Aqueous Phase	Concentration (%)	Organic Phase	Concentration (%)
OPD	2	TMC	0.1
MPD	2	TMC	0.1
PPD	2	TMC	0.1
MOPD	2	TMC	0.1
MMPD	2	TMC	0.1
MPPD	2	TMC	0.1
OPD/MOPD	1.5/0.5 1.0/1.0 0.5/1.5	TMC	0.1
MPD/MMPD	1.5/0.5 1.0/1.0 0.5/1.5	TMC	0.1
PPD/MPPD	1.5/0.5 1.0/1.0 0.5/1.5	TMC	0.1
MPD/2,6-MMPD	1.5/0.5 1.0/1.0 0.5/1.5	TMC	0.1
MOPD/MMPD	1.5/0.5 1.0/1.0 0.5/1.5	TMC	0.1
2,6-MMPD/MMPD	1.5/0.5 1.0/1.0 0.5/1.5	TMC	0.1

### 2.3. The Characterization of the RO Membranes

The cross-sectional structure and top morphology of the RO membranes were observed using a field emission scanning electron microscope (SEM, JEOLJSM-7800F, JEOL Ltd., Tokyo, Japan). The membrane samples were wetted with water, frozen in liquid nitrogen for a few minutes, and fractured. Both the top and cross-sectional areas were sputtered with gold prior to SEM observation. An atomic force microscope (AFM, BRUKER, Dimension Fastscan, Bruker Corporation, Billerica, MA, USA) was used to determine the surface roughness of the membrane using tapping mode in air; the roughness was depicted as mean roughness (R_a_) and root mean square (RMS). The scanning area was 4 µm^2^, and the values of RMS and R_a_ were averaged from three membrane samples.

### 2.4. The Desalination Performance

The water permeability and rejection ratio of the RO membranes were measured, respectively, by using 2000 ppm of NaCl aqueous solution in a cross-flow filtration system under the operating pressure of 1.5 MPa to evaluate the filtration performance of the polyamide RO membranes. The effective filtration area of the filtration cell was 26.4 cm^2^. The membrane was compacted with DI water for 3 h until the water flux remained steady. Then, a certain amount of NaCl was added to prepare the saline solution with a concentration of 2000 ppm to serve as a model of brackish water. The water permeation flux was determined through the direct measuring of the permeate volume collected over a certain period and calculated through Equation (1):

(1) $$J=\frac{V}{A\cdot t}$$

where J (L·m^−2^·h^−1^) is the permeation flux, *V* (L) is the volume of permeate, *A* (m^2^) is the effective membrane area, and *t* (h) is the collection time.

The salt rejection ratio was evaluated using Equation (2):

(2) $$R\;=\;(1-\frac{Cp}{Cf})$$

where *C_p_* (ppm) and *C_f_* (ppm) are the solute concentration of permeate and feed solutions, respectively. The salt concentration of feed and permeate solutions were determined by measuring the conductivity of the aqueous solutions using a conductivity meter (DDS-307A, Shanghai INESA Scientific Instrument Co., Ltd., Shanghai, China).

Chlorine resistances of the polyamide membranes were evaluated via the cross-flow filtration system with an aqueous solution of 100 ppm NaClO and 2000 ppm NaCl. The operating pressure was 1.5 MPa, and the temperature was controlled at 35–40 °C.

The antifouling properties of the polyamide membranes were determined using the cross-flow filtration system. The feed solution was a mixture of 100 ppm BSA and 2000 ppm NaCl, and the operating pressure and temperature were 1.5 MPa and 35–40 °C, respectively.

## 3. Results

### 3.1. The Morphology of the TFC Membranes

**Figure 2:**
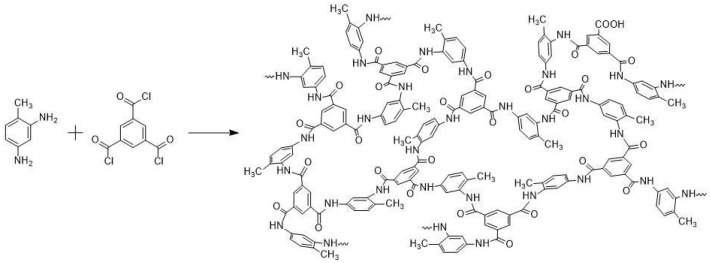
Schematic chemical structure of the barrier layer of the polyamide RO membrane fabricated from TMC and MMPD via interfacial polymerization.

Acyl chloride, TMC, isomeric phenylenediamines (i.e., OPD, MPD, and PPD), isomeric methyl-substituted phenylenediamines (i.e., MOPD, MMPD, 2,6-MMPD, and MPPD), as well as the integration of those monomers, were employed to fabricate the polyamide-based RO membrane by interfacial polymerization. The schematic polymerization of TMC and MMPD is shown in Figure 2.

A three-dimensional network was created through the condensation reaction of acyl chloride and amine groups. The cross-linking density of the polyamide matrix was kinetically controlled by the parameters of concentration of monomers, diffusion rate of monomers, hydrolysis of acyl chloride monomer, reaction time, and temperature [14]. It was believed that the packing density of the aromatic molecular structure in the polyamide barrier layer directly affected the water permeability and rejection ratio of the RO membrane [55]. Therefore, the different types of diamine monomers were employed to construct polyamide RO membranes and were studied.

**Figure 3:**
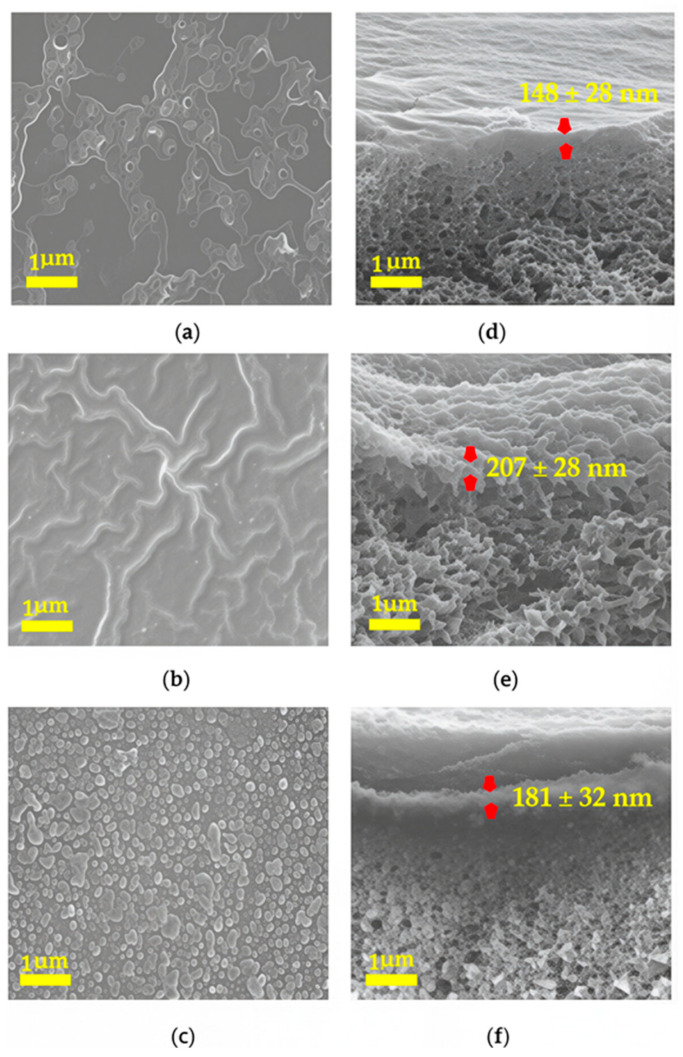
Top and cross-sectional views of the polyamide barrier layer formed through the reaction of TMC with OPD (**a**,**d**), MPD (**b**,**e**), and PPD (**c**,**f**). The magnification is 20,000×.

The surface and cross-sectional morphologies of polyamide barrier layers formed by interfacial polymerization of TMC and OPD, MPD, or PPD were investigated through SEM images, as shown in Figure 3.

The appearance of the surface morphology of the polyamide barrier layer was quite different with isomeric diamine monomers, even though the polymerization conditions for all monomers were the same. The surface of the OPD-based polyamide was distributed with collapsed vesicles, while that of the MPD-based polyamide exhibited a ridge-like structure. Both of them exhibited morphologies that were quite different from the morphology of the PPD-based polyamide, which has a connected particle-shaped pattern. One possible explanation for the formation of the crumple structure was that the exothermic nature of the reaction between acyl chloride and amine groups [56]. The released heat at the interface between water and organic solvent induced the evaporation of organic solvent and created a bubble-like structure on the surface of the polyamide. The morphology difference among the diamine isomeric monomers could be the result of the different diffusion rates of the diamine monomers from the aqueous phase to the organic phase and the partition coefficients between the two phases [57]. However, other than the surface morphology, the barrier layer thickness of different diamine-based polyamides remained at about 200 nm, as determined and marked from the cross-sectional images shown in Figure 3. Meanwhile, the surface roughness of the polyamide barrier layer was also different from the SEM images of cross-sectional views. The surface of the PPD-based polyamide was smoother than that of OPD- and MPD-based polyamide.

**Figure 4:**
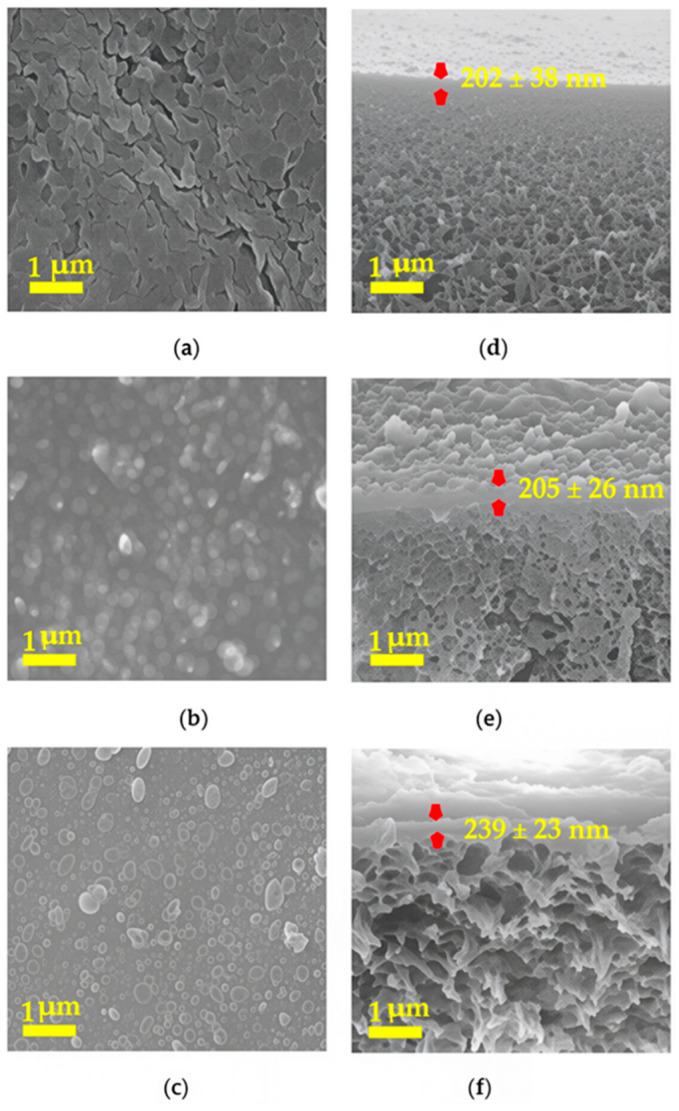
Top and cross-sectional views of the polyamide barrier layer formed through the reaction of TMC with MOPD (**a**,**d**), MMPD (**b**,**e**), and MPPD (**c**,**f**), respectively. The magnification is 20,000×.

To further investigate the effect of methyl-substituted diamine monomers on the surface morphologies of corresponding polyamides, SEM images were collected as shown in Figure 4.

It can be seen that the surface morphologies of methyl-substituted MOPD, MMPD, and MPPD-based polyamide barrier layer were composed of all collapsed vesicles; however, the vesicle sizes of the MMPD- and MPPD-based polyamide exhibited a broad distribution, while that of MOPD-based polyamide appeared more crumpled. Compared with that of diamine monomers without methyl-substituted groups, the surface of methyl-substituted diamine-based polyamides was smoother and more even. This revealed that the methyl group located on the adjacent site of the amine on the benzene ring exhibited distinguished effects on the surface structure and morphologies of the resulting polyamides.

The term “topological index” was employed to describe the structure difference in the isomeric monomers, and the relationship of topological indexes versus partition coefficients of benzene and its derivatives between water and hexane was established. The partition coefficients of isomeric diamine monomers could be evaluated from the linear dependence of partition coefficients and topological indexes.

**Figure 5:**
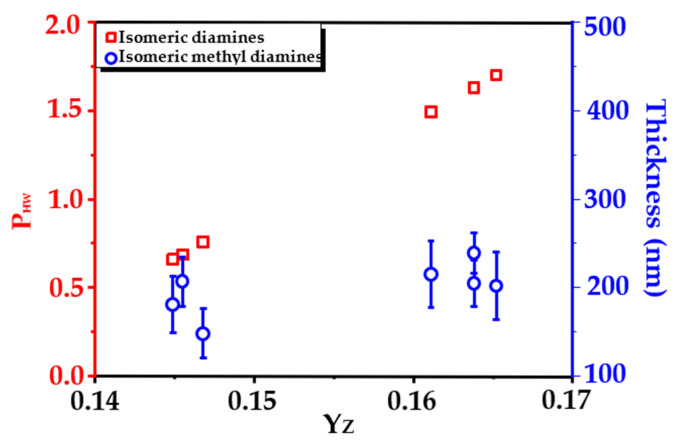
The relationships of the partition coefficients of isomeric diamine monomers between water and hexane, their topological indexes, and the polyamide barrier layer thicknesses.

Meanwhile, the barrier layer thickness of polyamides formed by interfacial polymerization was also related with the topological index. The correlation among the partition coefficients, polyamide barrier layer thicknesses, and the corresponding topological indexes were plotted, as shown in Figure 5.

It can be seen that the partition coefficient of diamine monomers with methyl substitution between hexane and water is about one more logarithmic point than that of diamine monomer without methyl groups, that is, the methyl group makes the diamine easier to diffuse into the organic phase. As a result, the barrier layer formed by interfacial polymerization of methyl-substituted monomers was thicker than that formed by interfacial polymerization of monomer without methyl groups, as shown in Figure 5, verified by measurements of the cross-sectional SEM images of polyamide barriers.

**Figure 6:**
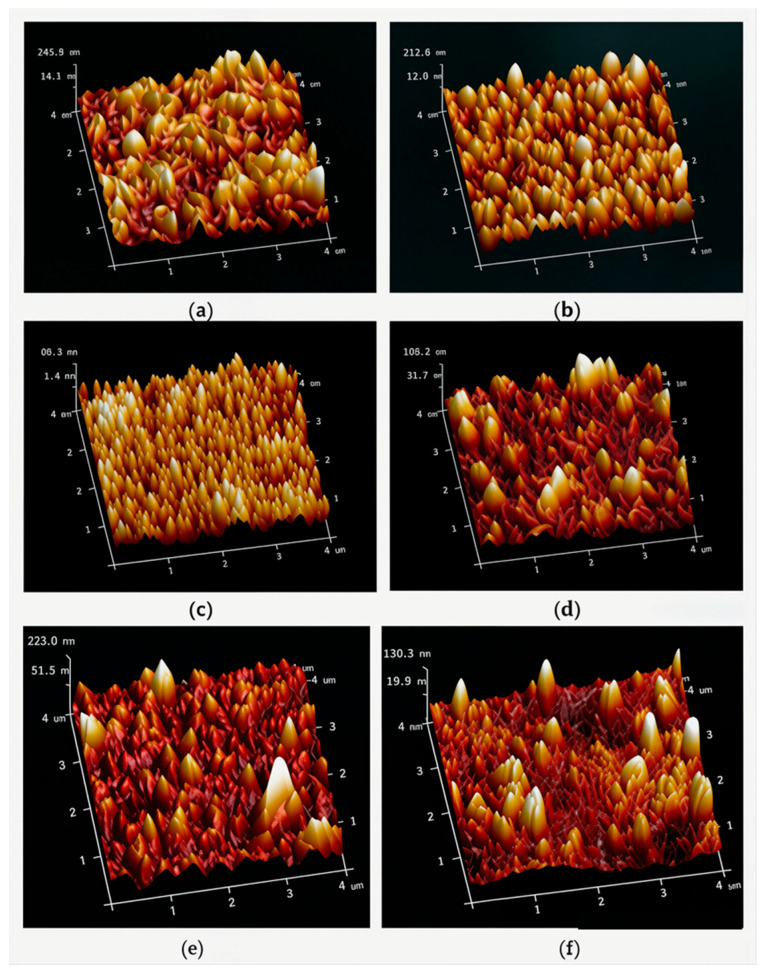
3D AFM images of the surface morphologies of the polyamides prepared with OPD (**a**), MPD (**b**), PPD (**c**), MOPD (**d**), MMPD (**e**), and MPPD (**f**).

The surface roughness of the polyamide barriers could be determined quantitatively through AFM measurements, as shown in Figure 6.

The surface roughness of the polyamide barrier layers was determined quantitatively by AFM using tapping mode in air. It was very interesting that the surface roughness of polyamides varied with the isomeric diamine monomers of OPD, MPD, and PPD, while PPD exhibited the smoothest surface morphology, as shown in Figure 6c. Also, the surface roughness became significantly smoother when methyl-substituted diamine monomers, i.e., MOPD, MMPD, and MPPD, were used to fabricate the polyamide barrier layers, instead of OPD, MPD, and PPD, respectively. Both the mean roughness (Ra) and root mean square (RMS) were employed to describe the surface morphologies quantitatively, as shown in Table 2.

A possible reason for the low surface roughness of the polyamide barrier layer fabricated with methyl-substituted diamine monomers could be the lower exothermal enthalpy difference generated in the interfacial polymerization process or the lower mobility of the monomers due to the relative reactivity when compared with that of the diamine monomers without substitution [56,58]. These results indicated that the methyl group located on the benzene rings certainly affected the morphologies of the corresponding polyamide barrier layers due to the reactivity. Meanwhile, this also changed the separation efficiency of the RO membranes, as evidenced by the desalination performance in the following section.

### 3.2. The Desalination Performance of the RO Membranes

**Figure 7:**
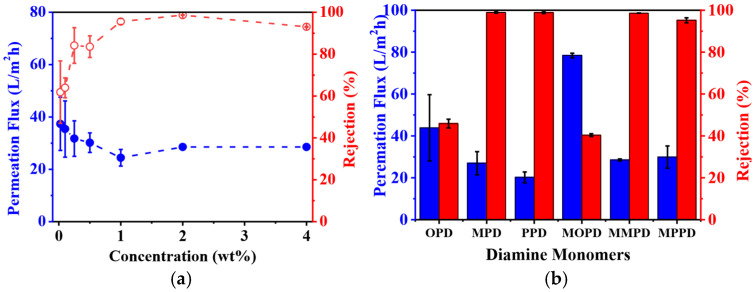
The effect of MMPD concentration on permeation flux and rejection ratio of the MMPD-based polyamide RO membrane (**a**) and the desalination performance of the polyamide RO membranes based on different isomeric monomers (**b**).

The methyl-substituted *m*-phenylenediamine monomer, MMPD, and acyl chloride monomer, TMC, were employed to fabricate the polyamide-based RO membrane with the PS-35 UF substrate as the support combined through interfacial polymerization. In order to investigate how the concentration of MMPD affects the desalination performance of the RO membrane in terms of permeation flux and rejection ratio, a variety of MMPD aqueous solutions with different concentrations were used in the interfacial polymerization process where the concentration of TMC in hexane remained the same. The desalination performance of the RO membranes was demonstrated as shown in Figure 7a.

The permeation flux of the RO membrane decreased with the increase in the concentration of MMPD within the range of 0.025–4.0 wt%; as expected, the rejection ratio of the membrane increased essentially to 98.6 ± 0.1% when the concentration of MMPD reached 2.0 wt%. At a low concentration of MMPD, the cross-linking degree between MMPD and TMC was low due to the excess of TMC; as a result, the aromatic molecular structure was very loose, thereby causing the high permeation flux and low rejection ratio as well. With increasing the concentration of MMPD, the diffusion rate of MMPD through the interface increased and more MMPD and TMC molecules met together to create a dense cross-linking structure in the interfacial polymerization process. Thus, the corresponding RO membrane exhibited a high rejection ratio against NaCl with decreased permeation flux, as shown in Figure 7 [59]. Further increases in the concentrations of MMPD caused the rejection ratio to decrease to 93.0 ± 0.2%, which could be attributed to the lack of sufficient TMC, resulting in cross-linking defects [60]. Therefore, the optimized concentration of MMPD was 2.0 wt%, while the permeation flux of the membrane was 28.5 ± 0.5 L/m^2^ h; the rejection ratio was as high as 98.6 ± 0.1% against 2000 ppm of NaCl aqueous solution.

Encouraged by this finding, we chose six isomeric phenylenediamine monomers, i.e., OPD, MPD, PPD, MOPD, MMPD, and MPPD, to use in fabricating polyamide-based RO membranes where the concentrations of diamines and TMC were 2.0 wt% and 0.1 wt%, respectively. The desalination performance was demonstrated to investigate the correlation between the isomeric structure of diamine monomers and the filtration efficiency of the RO membranes, as shown in Figure 7b.

It was predicted that the relative location of the two amines, as well as the methyl group on the aromatic molecular structure, would essentially affect the filtration efficiency of the RO membranes [52]. Both the MPD- and PPD-based polyamide RO membranes exhibited rejection ratios higher than 98.8%, and their permeation fluxes are 27.0 ± 5.5 and 20.2 ± 2.6 L/m^2^ h, respectively, indicating that those membranes were suitable for the desalination, though the permeation flux of the MPD-based RO membrane was higher than that of the PPD-based one. However, the OPD-based RO membrane exhibited a permeation flux and rejection ratio of 40.3 ± 12.8 L/m^2^ h and 50.1 ± 6.2%, respectively, under the same conditions, which cannot be used practically for the desalination. Therefore, it was concluded that the rejection ratio of the RO membranes was drastically affected by the relative location between the two amines of isomeric monomers, where the meta- and para-position may release steric hindrance of the aromatic molecular structure and offer high rejection ratio to the corresponding RO membranes. As a comparison, OPD, with its adjacent amine groups, was unable to react with two TMC monomers simultaneously due to the steric hindrance, which retarded the formation of cross-linking structure of polyamide, and, therefore, the OPD-based polyamide RO membrane exhibited low rejection ratio against sodium chloride, as shown in Figure 7b.

Furthermore, the methyl substituent, which replaced the hydrogen located on the ortho position of the amino group of the monomers, may also affect the filtration efficiency of the corresponding RO membranes. It was seen that both MPD- and MMPD-based polyamide RO membranes have similar permeation fluxes of 27.0 and 28.5 L/m^2^ h and a rejection ratio of above 98.6%. In other words, the methyl group located on the benzene ring of MMPD does not obviously change the packing density of the aromatic molecular structure. However, the MPPD-based RO membrane exhibited a rejection ratio of 96.5%, which was 2.1%-lower than that of the PPD-based RO membrane, having a rejection ratio as high as 98.8%. The difference between MPPD and PPD revealed that the methyl group on the aromatic molecular structure certainly affects the rejection ratio of the corresponding RO membranes. On the other hand, the permeation flux of MPPD-based polyamide RO membrane, which was 24.2 L/m^2^ h, was higher than the 20.2 L/m^2^ h permeation flux of the PPD-based membrane.

A possible explanation for this could be the configuration of the methyl group, which tilted out of the plane of MPPD with an orthogonal configuration [61]. The orthogonal configuration will occupy more space, thereby reducing the packing density of the aromatic molecular structure. As a result, the size of the molecular channels for water molecule transportation could be larger in MPPD-based polyamide, and, therefore, the rejection ratio of polyamide barrier layer could be lower than that of the polyamide barrier layer without methyl groups. This could be further evidenced by the investigation of MOPD-based polyamide RO membrane, which exhibited decreased rejection ratio of 40.4% when compared with 50.1% of the OPD-based RO membrane. However, it was very interesting that the permeation flux of the MOPD-based polyamide RO membrane was 78.4 ± 1.1 L/m^2^ h, which was about twice that of the permeation flux of the OPD-based RO membrane (43.9 ± 15.8 L/m^2^ h). Therefore, it was confirmed once again that the methyl groups drastically affect the permeation flux and rejection ratio of the RO membranes through changing the packing density of the aromatic molecular structure in the polyamide barrier layer.

### 3.3. Molecular Dynamic Simulation

The molecular models of six monomers were constructed in Materials Studio 2017 (MS 2017). We hypothesized that the configurational monomers have energy-minimized conformations at steady state (Geometry Optimization: fine quality, 5000 iterations), which could be achieved based on the calculation using the Forcite method in a COMPASS II field, as shown in Table 3.

It was very interesting that the MPD monomer exhibited the lowest potential energy, reflecting the highly thermodynamic stability of the MPD structure, which was the most suitable for constructing an RO membrane for the desalination. The introduction of the methyl substituent increased the potential value of the repeating unit from −31.41 to −22.46 kcal/mol, implying that the methyl group certainly affected the thermodynamic stability of the MMPD structure. Furthermore, PPD and MPPD monomers have modest potential energies of −28.92 and −22.08 kcal/mol, respectively, and the corresponding membranes are still usable for desalination. However, OPD and MOPD monomers exhibited high potential energies of 0.15 and 7.45 kcal/mol, which could be attributed to the crowded conformation of two adjacent amino groups, and the corresponding RO membranes failed for use in desalination with low rejection ratios. Therefore, it was concluded that the total potential energy could be used as the criteria for judgment of whether a configurational monomer is suitable for RO membrane fabrication.

It is interesting that a tilted angle, from 2.4° to 2.9°, was observed after the introduction of the methyl substituent on the neighboring position of the amine group on the aromatic molecular ring, indicating that the configuration of the repeating unit was essentially changed; as a result, the corresponding aromatic molecular structure could be rearranged, and, therefore, the separation efficiency, including the permeation flux and rejection ratio, would be affected by the methyl groups. In particular, the MOPD-based repeating unit exhibited a crowded conformation where a methyl group was adjacent to two amine groups. The distortion of the structural unit connected to adjacent diamines of MOPD may decrease the packing density of the aromatic molecular structure where water molecules can go through the barrier layer easily. Thus, it was concluded that the methyl substituent next to the diamine groups could increase the permeation flux of the corresponding RO membrane.

### 3.4. Desalination Performance of RO Membrane with Integrated Diamine Monomers

**Figure 8:**
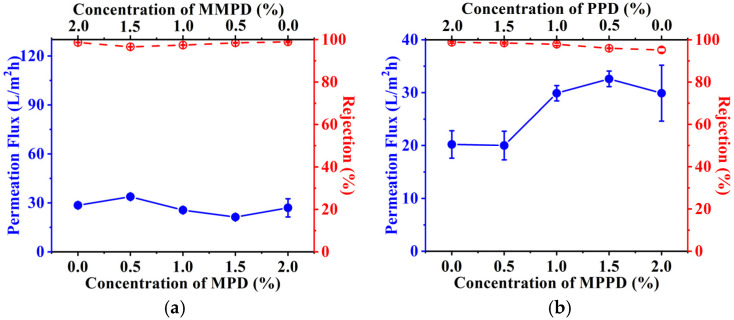

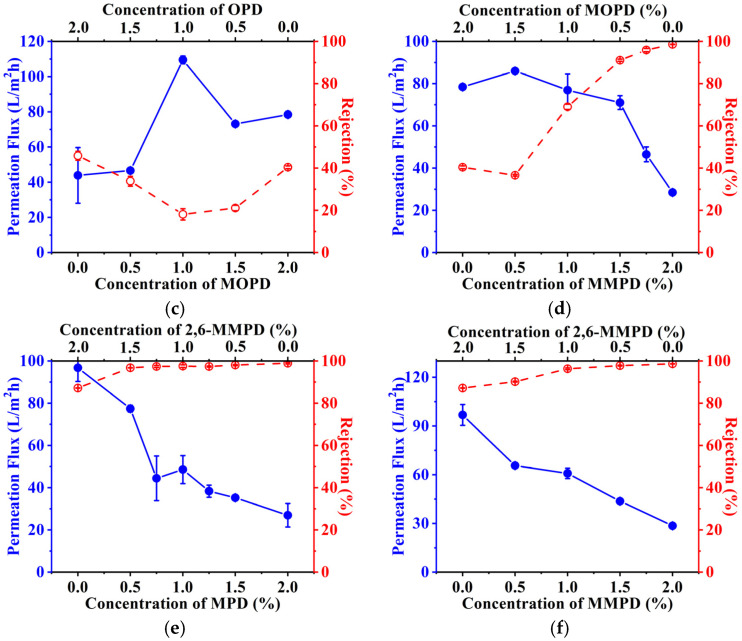
The diagrams of the desalination efficiency of the RO membranes vs. aqueous phase compositions of MPD and MMPD (**a**), PPD and MPPD (**b**), OPD and MOPD (**c**), MOPD and MMPD (**d**), MPD and 2,6-MMPD (**e**), and MMPD and 2,6-MMPD (**f**), respectively.

The molecular dynamic simulation revealed that the integrational monomers can improve the permeability of the RO membrane by changing the packing density of the aromatic molecular structure [62,63,64]. To further experimentally verify the simulation results, RO membranes based on two integrated diamine monomers were fabricated, and their desalination performances were investigated using a cross-flow filtration system, as shown in Figure 8.

As expected, the permeation flux and rejection ratio of the RO membrane based on MPD and MMPD remained almost unchanged throughout their ratio ranges, as shown in Figure 8a, indicating that the integration of MPD and MMPD does not essentially change the structure of the polyamide barrier layer, and, therefore, the permeation flux and rejection ratio remained as 27.2 L/m^2^ h and 97.5%, respectively.

It is very interesting that the permeation flux of the RO membrane increased with the increase in the ratio of MPPD to PPD, indicating that the methyl substituent of MPPD can decrease the packing density of the aromatic molecular structure in the barrier layer, which remarkably increases the permeation flux of the corresponding RO membrane, as shown in Figure 8b. The permeation flux of the RO membrane with the equal ratio of PPD to MPPD was 1.5-times higher than that of PPD-based membrane; meanwhile, the rejection ratios of the MPPD- and PPD-based RO membranes remained 98.0%. Increasing the ratio of MPPD further, the rejection ratio of the membrane decreased to 96.5%, while the permeation flux of the membrane increased to 32.6 L/m^2^ h. Similar trend was observed for OPD and MOPD-based RO membranes, wherein the permeation flux increased drastically with the increasing ratio of MOPD in the integrated monomers, as shown in Figure 8c. Unsurprisingly, the integration of OPD and MOPD-based RO membrane resulted in poor desalination performance, with the rejection ratio between 18.1% and 58.6%, due to the steric hindrance of methyl and two amino groups of each monomer.

Furthermore, we believe that MMPD-based aromatic molecular structure can retain a high rejection ratio for the RO membrane, while the MOPD-based structure can provide a high permeation flux. The integration of two types of monomers may offer the RO membrane the high permeation flux and reasonably high rejection ratio simultaneously. To verify this hypothesis, two different monomers, MMPD and MOPD, were employed to construct an integrated polyamide barrier layer, and the desalination performance of the RO membranes based on different ratios of MMPD to MOPD were investigated, as shown in Figure 8d.

As expected, the permeation flux of the RO membrane increased with the concentration of MOPD and decreased with that of MMPD owing to their different configurations of monomers, which created integrated aromatic molecular structure for water molecule transportation. The highest permeation flux was 46.5 L/m^2^ h when the concentration of MOPD was 0.25%, which was 1.6-times higher than that of the pure MMPD-based RO membrane, while the rejection ratio remained 96.0%.

To further explore the effect of the methyl substituent, 2,6-diaminetoluene (2,6-MMPD) (where the methyl group is located between the two amino groups) was employed and coupled with MPD and MMPD, respectively, to form an integrated aromatic molecular structure in the barrier layer of the RO membrane. The desalination performance of the RO membranes was demonstrated as shown in Figure 8e,f. The permeation flux and rejection ratio of the pure 2,6-MMPD-based RO membrane was 117.8 L/m^2^ h and 67.8%, respectively, indicating that the membrane failed to be used for the desalination. A molecular simulation revealed that the total potential energy of 2,6-MMPD monomer was −3.59 kcal/mol, which indicated that the crowded conformation of the amino and methyl groups in the monomer 2,6-MMPD decreased packing density of the aromatic molecular structure where high permeation flux and low rejection ratio in desalination was obtained. However, it was interesting to note that the integration of two isomeric monomers, either MPD/2,6-MMPD or MMPD/2,6-MMPD, offered high permeation flux and high rejection ratio to the RO membrane. The permeation fluxes of the RO membranes based on MPD/2,6-MMPD and MMPD/2,6-MMPD, respectively, were 48.6 and 60.8 L/m^2^ h, which is two times higher than that of the conventional RO membrane (27.1 L/m^2^ h); the rejection ratios remained higher than 97.6%. Again, the high permeation flux and high rejection ratio of the RO membrane was attributed to the methyl group-induced integrated aromatic molecular structure in the polyamide barrier layer of the RO membrane.

**Figure 9:**
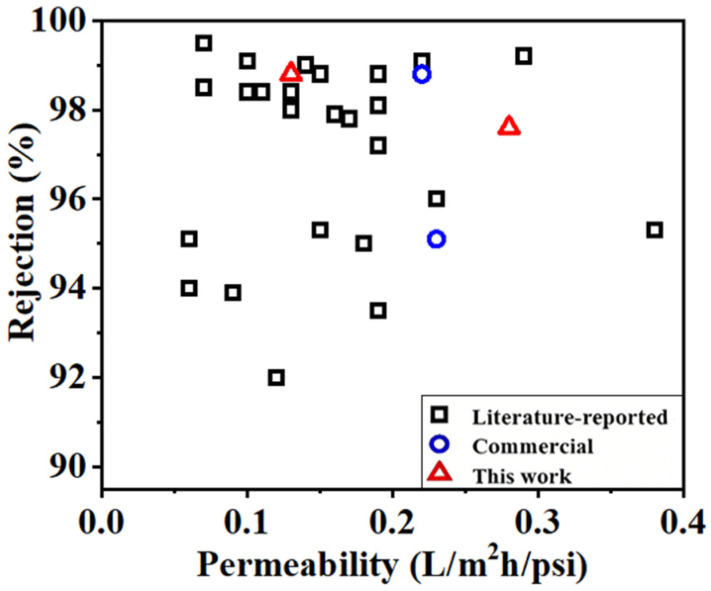
Desalination efficiency comparisons of the RO membranes with integrated diamine monomers and other lab-made and commercially available RO membranes [66,67,68,69,70,71,72,73,74,75,,76,77,78,79,80,81,82,83,84,85,86,,87,88,89].

Finally, the performance of the RO membrane based on integrated diamine monomers were compared with commercially available counterparts and other lab-made RO membranes reported in the literature [65,66,67,68,69,70,71,72,73,74,75,76,77,78,79,80,81,82,83,84,85,86,87,88], as shown in Figure 9, the detailed data are listed in Table 4. It was clear that the permeation flux of most RO membranes in the previous literature was limited to 0.2 L/m^2^ h/psi, while the rejection ratios ranged from 94.0% to 99.5%. Thus, the RO membranes based on integrated diamine monomers exhibited higher permeation flux than most of those reported in previous works and commercially available RO membranes while maintaining a comparable rejection ratio. Those results indicated that the RO membranes fabricated by the integrated diamine monomers could be potentially used in the desalination processes for brackish water and seawater with high separation performance.

**Table 4:** Membrane performance reported in the literature and in this work.

TFC Membrane	NaCl Rejection (%)	Permeance (L/m^2^ h/psi)	Reference
MPD-BHAC	99.1	0.10	[66]
MPD-BTAC	98.8	0.19	[66]
MPD-BPAC	99.0	0.15	[66]
MPD-TMC	94.0	0.06	[67]
MPD-TMC (DMSO)	99.2	0.29	[68]
MPD-TMC	98.4	0.11	[70]
MMPD–CFIC@CFIC–DMMPD	95.3	0.15	[73]
MMPD/DMMPD–CFIC	96.0	0.23	[73]
MeO-PEG-MPD	93.5	0.19	[74]
MPD-BTC-TMC	98.8	0.07	[75]
MPD-TMC	99.0	0.07	[78]
MPD-mm-BTEC	98.4	0.10	[79]
MPD-om-BTEC	97.8	0.17	[79]
MPD-op-BTEC	97.2	0.19	[79]
GO-MPD-TMC	99.3	0.08	[80]
MPD-TMC-NaA	98.0	0.13	[84]
MPD-TMC	99.0	0.14	[85]
GO-MPD-TMC	95.7	0.18	[86]
NaA-MPD-TMC	93.9	0.09	[32]
Si-MPD-TMC	95.1	0.06	[87]
MPD-TMC-NPs	97.9	0.16	[88]
S-BAPS + TMC	99.6	0.22	[89]
MPD-TMC	98.8	0.13	This work
MPD/2,6-MMPD-TMC	97.6	0.28	This work

### 3.5. The Contact Angle of the RO Membrane

**Figure 10:**
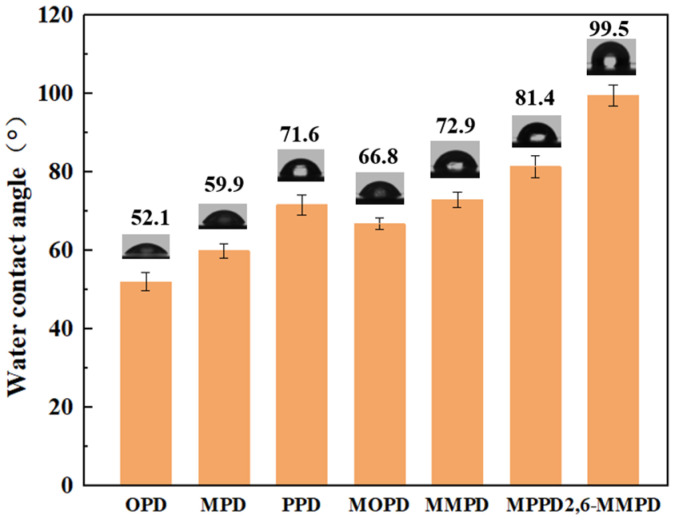
The water contact angles of the seven polyamide membranes.

Surface wettability is a critical characteristic of membranes because it significantly affects both filtration performance and fouling behavior [90,91]. The hydrophilicity of polyamide membranes prepared from seven diamine monomers was characterized by water contact angle measurements, as shown in Figure 10.

The polyamide membrane based on 2,6-MMPD exhibited the highest water contact angle (~99.5°) among all samples, whereas the other six membranes all exhibited water contact angles of less than 90°. Furthermore, the polyamide membranes prepared with methyl-substituted diamine monomers generally exhibited larger water contact angles compared to those based on non-methyl-substituted diamine monomers—indicating that the surfaces of the latter were more hydrophilic. This observation was in agreement with the subsequent antifouling properties of the membranes. These results were also consistent with the AFM observations, which showed that rougher membrane surfaces—such as the OPD-, MPD-, and PPD-based membranes—displayed more pronounced hydrophilicity, while MOP-, MMPD-, and MPPD-based membranes were more hydrophobic. This aligned with the expected relationship between surface roughness and hydrophilicity for such materials.

### 3.6. The Chlorine Resistance of the RO Membrane

**Figure 11:**
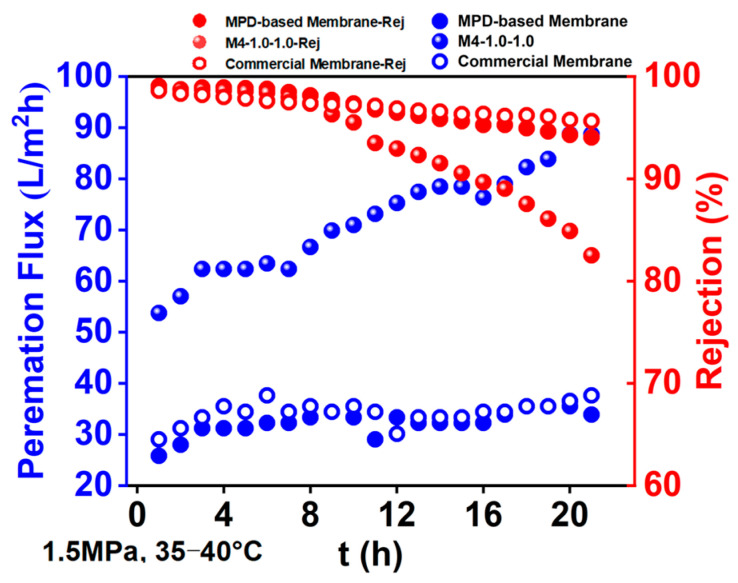
The chlorine resistance of the MPD/2,6-MMPD, MPD-based, and commercial RO membranes.

The chlorine resistance of the MPD/2,6-MMPD- and MPD-based RO membrane, as well as the commercially available RO membrane (LC HR-4040), in aqueous solution of 100 ppm NaClO and 2000 ppm NaCl was evaluated through a cross-flow filtration system, as shown in Figure 11.

As can be seen from the figure, with the increase in filtration time, the retention rate of the three membranes kept a slow decline, while the water flux increased. The possible reason is that as the system operates, the water temperature continues to rise, resulting in an increase in water flux. When it reached 3 h, the temperature of the filtration system was stable at 35~40 °C. When the filtration time reached 8 h, the retention rate of the three membranes began to decline, but the retention rate of MPD/2,6-MMPD-based dropped the most sharply, from 98.0% at 7 h to 82.5% at 21 h. The water flux increased sharply from 62.4 L/m^2^ h in 7 h to 88.7 L/m^2^ h in 21 h. On the other hand, the retention rate of MPD-based reverse osmosis membrane and commercial membrane decreased relatively slowly, from 98.5% and 97.5% in 7 h to 94.0% and 95.7% in 21 h, respectively, and the water flux basically remained at about 33.3 L/m^2^ h.

It can be seen that the MPD/2,6-MMPD-based membrane shows weak chlorine resistance compared to conventional RO membranes and commercial RO membranes. The possible reason is that the mixed monomer does not modify MPD in nature, and the other aqueous monomer (2,6-MMPD) does not have the property of being more easily replaced by chlorine in the resulting polyamide film, which plays a protective role in the polyamide layer. On the contrary, with the addition of 2,6-MMPD, the packing density of the polyamide layer changes and the free volume is larger, which provides more attack sites for active chlorine (originally affected by the densification of polyamide, which may be limited to attack on the surface), so MPD/2,6-MMPD-based membrane shows weaker chlorine resistance.

### 3.7. The Antifouling Properties of the RO Membrane

**Figure 12:**
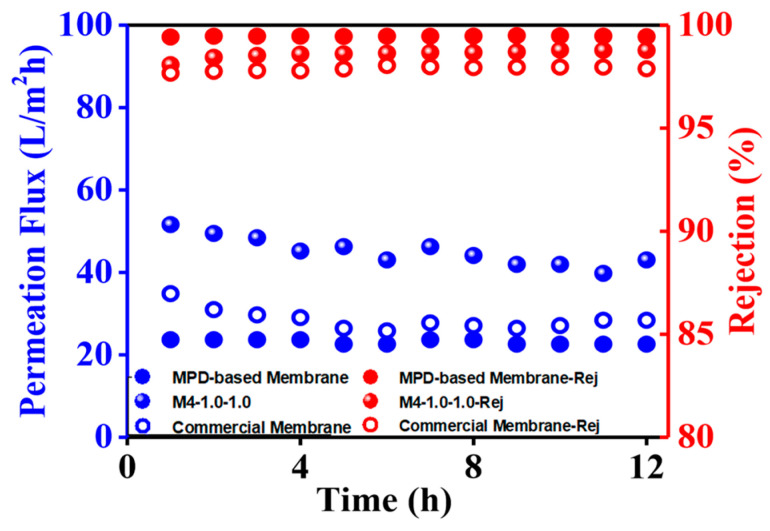
The desalination performance of the MPD/2,6-MMPD, MPD-based, and commercial RO membranes.

The antifouling properties of the MPD/2,6-MMPD- and MPD-based RO membrane, as well as the commercially available RO membrane (LC HR-4040), was evaluated by employing BSA, a commonly used protein foulant, with 2000 ppm of NaCl as the feeding solution, through a cross-flow filtration system, as shown in Figure 12.

It can be seen that the permeation flux of the MPD-based membrane remained almost unchanged at 23.7 L/m^2^ h, while the rejection ratio was 99.4%, indicating that the MPD-based RO membrane has good antifouling properties within the testing period. Both the MPD/2,6-MMPD-based and commercial RO membrane exhibited higher permeation fluxes of 43.0 and 26.5 L/m^2^ h, though a flux decay of 16.7% and 24.1% was observed. The flux decay could be attributed to the surface hydrophobicity of the RO membrane, while the water contact angles of MPD/2,6MMPD-based and commercial RO membranes were 58.3° and 43.7°, respectively, which is lower than 57.0 of the MPD-based RO membrane. Nevertheless, the permeation flux of the MPD/2,6MMPD-based RO membrane was retained at 43.0 L/m^2^ h after 12 h desalination performance which was about two times higher than that of the MPD-based and commercial membrane under the same conditions.

### 3.8. Long-Term Desalination Performance

**Figure 13:**
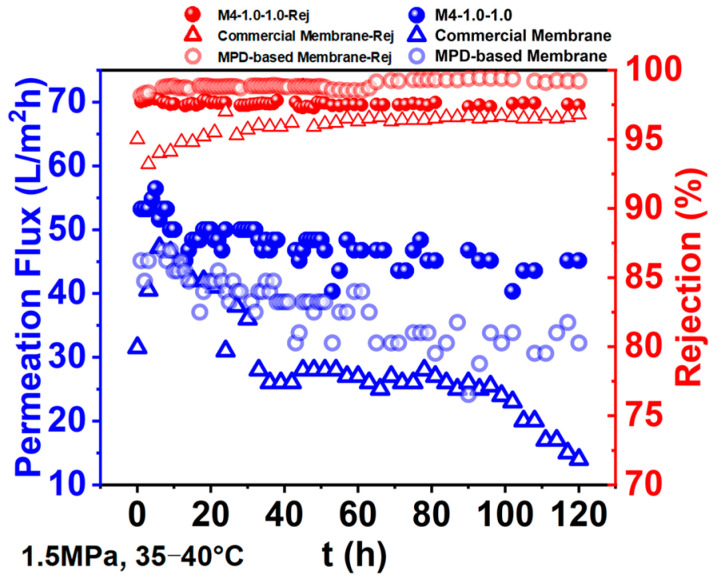
Long-term desalination performance of MPD/2,6-MMPD, MPD-based, and commercial RO membranes.

A long-term desalination performance was demonstrated to evaluate the potential application of the MPD/2,6-MMPD-based RO membrane, as well as MPD-based and commercially available membranes, where 2000 ppm of NaCl aqueous solution was employed as the feed solution. The operating pressure was kept at 1.5 MPa, and the temperature was controlled in the range of 35–40 °C. The variation in the permeation flux and rejection versus time was plotted, as shown in Figure 13.

The permeation flux of the MPD/2,6-MMPD-based membrane decreased at very beginning and then retained stable throughout 120 h-desalination performance. The final desalination efficiency, in terms of permeation flux and rejection ratio, were 45.2 L/m^2^ h and 32.3%, respectively, which was 1.4 times higher than that of the MPD-based membrane after 120 h desalination. For comparison, the permeation flux of the commercial membrane decreased continuously and approached 34.0 L/m^2^ h, which was about 1.3 times lower than that of the MPD/2,6-MMPD-based RO membrane, indicating the great advantages for the long-term desalination performance. Meanwhile, the rejection ratio of the MPD-based RO membrane remained at 98.2–99.2% throughout the desalination performance. It was interesting that the rejection ratio of the MPD/2,6-MMPD-based RO membrane was 97.6%, which was higher than 96.8%, the rejection ratio of the MPD/2,6-MMPD RO membrane, though a 1.3-times higher permeation flux was achieved, implying the potential application of the MPD/2,6-MMPD RO membrane for industrial desalination.

## 4. Discussion

A variety of RO membranes with different polyamide barrier layers have been fabricated successfully, where phenylenediamine, methyl-substituted phenylenediamine, and the integration of isomeric monomers were employed to explore the relationship between the aromatic molecular structure of the polyamide barrier layer and the desalination efficiency of the corresponding RO membranes. The surface morphologies of RO membranes were investigated via the SEM and AFM measurements, where we found that the location of amino and methyl groups remarkably affect the morphologies of the polyamide barrier layer, which could be attributed to the steric hindrance, configuration, and partition coefficient of different diamine monomers. As a result, the surface roughness of methyl-substituted diamine-based polyamide was much lower than that of polyamide from the diamine without methyl substitution. Further investigation of the polyamide RO membranes were carried out through the evaluation of the filtration efficiency for the desalination performance. We found that the permeation flux and rejection ratio of the RO membrane drastically depended on the structure of the polyamide barrier layer, i.e., the packing density of the aromatic molecular structure, while loose packing offers high permeation flux and relatively lower rejection ratios. The introduction of methyl groups on the benzene rings could adjust the packing density of the aromatic molecular structure and, therefore, could adjust the permeation flux and rejection ratio of the RO membrane. The MMPD-based polyamide RO membrane, where methyl groups were involved on the aromatic molecular structure, exhibited a high rejection ratio of 98.6 ± 0.1% and can be used for the desalination of brackish water and seawater. The effects of amino and methyl groups were explored through a molecular dynamic simulation of the configurational monomers, where the crowd conformation and low packing density of the aromatic molecular structure offer high permeation flux in the membrane. Moreover, the RO membranes with integrated diamine monomers, such as MMPD/2,6-MMPD and MPD/2,6-MMPD, enhanced the permeation flux further by improving the polyamide molecular structure. As evidence, the optimized MPD/2,6-MMPD-based RO membrane exhibited about two times higher permeation flux than that of pristine and commercial RO membranes, while the rejection ratio remained as high as 97.6%. The antifouling properties and long-term desalination performance of MPD-2,6-MMPD-based RO membrane was evaluated, where the permeation flux was about twice that of the MPD-based and commercially available RO membrane, while the rejection ratio remained high, which indicated that the integration of the polyamide molecular structures in the barrier layer offers high desalination performance in the RO membranes.

## Figures and Tables

**Table 2:** Surface roughness of the polyamide barrier layers of the RO membranes.

Monomer Composition	RMS (nm)	R_a_ (nm)
OPD/TMC	65.7 ± 6.2	51.2 ± 4.0
MPD/TMC	64.0 ± 10.0	52.2 ± 7.5
PPD/TMC	24.7 ± 0.5	18.9 ± 1.7
MOPD/TMC	36.5 ± 3.3	27.9 ± 2.0
MMPD/TMC	45.1 ± 2.7	31.1 ± 0.9
MPPD/TMC	31.1 ± 2.5	23.8 ± 1.8

**Table 3:** Simulation results of configurational monomers.

Configurational Monomers	Total Potential Energy (kcal/mol)	Methyl-Tilted Angle (°)
OPD	0.15	-
MPD	−31.41	-
PPD	−28.92	-
MOPD	7.45	2.9
MMPD	−22.46	2.4
MPPD	−22.08	2.5

## Data Availability

The original contributions presented in this study are included in the article. Further inquiries can be directed to the corresponding author.
